# Wi-Fi-Based Effortless Indoor Positioning System Using IoT Sensors

**DOI:** 10.3390/s19071496

**Published:** 2019-03-27

**Authors:** Muhammad Usman Ali, Soojung Hur, Yongwan Park

**Affiliations:** Department of Information and Communication Engineering, Yeungnam University, Gyeongsan, Gyeongbuk 38541, Korea; musmanali@ynu.ac.kr (M.U.A.); sjheo@ynu.ac.kr (S.H.)

**Keywords:** indoor positioning, IPS, fingerprinting, Wi-Fi radio maps, path loss maps, RSSI, IoT sensors

## Abstract

Wi-Fi positioning based on fingerprinting has been considered as the most widely used technology in the field of indoor positioning. The fingerprinting database has been used as an essential part of the Wi-Fi positioning system. However, the offline phase of the calibration involves a laborious task of site analysis which involves costs and a waste of time. We offer an indoor positioning system based on the automatic generation of radio maps of the indoor environment. The proposed system does not require any effort and uses Wi-Fi compatible Internet-of-Things (IoT) sensors. Propagation loss parameters are automatically estimated from the online feedback of deployed sensors and the radio maps are updated periodically without any physical intervention. The proposed system leverages the raster maps of an environment with the wall information only, against computationally extensive techniques based on vector maps that require precise information on the length and angles of each wall. Experimental results show that the proposed system has achieved an average accuracy of 2 m, which is comparable to the survey-based Wi-Fi fingerprinting technique.

## 1. Introduction

The precise indoor location information of a mobile node with ubiquitous resources in an environment is one of the hot topics in the field of geolocation technologies today. Global Navigation Satellite Systems (GNSS) such as Global Positioning System (GPS) can only be used for outdoor location-based services (OLBS), while for indoor environments, satellite signals are strongly attenuated because of multipath effects, etc. and become unreliable for positioning indoors. As a result, many IPS solutions have been proposed so far to enable indoor location-based services (ILBS) for complex indoor environments, including hospitals, airports, shopping malls, and so on. ILBS also helps with resource management, which includes optimal deployment, locating, and monitoring of resources. IPS solutions are classified as infrastructure-based and infrastructure-free technologies. Infrastructure-based solutions primarily include Radio Frequency Identifier (RFID), RF, Bluetooth, and Ultra-Wideband (UWB) based technologies, while infrastructure-free solutions are typically based on Wi-Fi, magnetic field, motion sensors (Inertial Measurement Unit (IMU)) and vision techniques [1]. Infrastructure-free solutions are preferred because of their low cost and ease of use as compared to the infrastructure-based solutions that require costly pre-installation and configuration of specialized hardware in the environment.

Wi-Fi-based techniques are generally divided into calibration techniques and techniques without calibration [2]. An indoor environment is a hybrid environment because of the coexistence of the cases of Line-of-sight (LOS) and NON-line-of-sight (NLOS). This complexity in an indoor environment restricts the use of calibration-free techniques such as trilateration or triangulation. As a result, IPS solutions using the time of arrival (TOA) and time difference of arrival (TDOA) [3] suffer from a propagation error on several paths. In addition, the synchronization of receiver/transmitter-side times and the accuracy of short-range flight time measurements make them more difficult. The angle of arrival (AOA) technique, on the other hand, requires complex hardware for angle calculation. Position estimation using the value of the received signal strength indicator (RSSI) is a solution to avoid time synchronization problems. The Wi-Fi fingerprinting is a calibration-based technique that uses the RSSI value of APs received at a particular location. Fingerprinting is one of the most promising techniques for positioning inside a Wi-Fi environment. In addition, it is independent of the requirements for indoor maps, as well as the location of transmitters and multipath effects [4]. Fingerprinting requires pre-deployment survey of the environment, called the offline training phase, and constitute a calibrated RSSI database received at specific reference points. This calibration database is then used in the estimation of the position, called the online phase of fingerprint matching. Calibration of the fingerprinting database through the survey is a laborious task and limits the widespread adoption of this technique [5]. Numerous solutions have been proposed so far to reduce the intensity of the efforts deployed in the calibration phase of the database. These solutions mainly include crowd-sourcing techniques [6,7,8,9], interpolation to sparse reference points, and so on [10,11]. In contrast, a calibration-free path loss model technique using the map and location information of APs is also proposed in [12] to avoid the tedious task of site survey required by calibration of the Wi-Fi fingerprint database. Similarly, many other variants of fingerprinting techniques have been proposed so far. However, the main problems, namely the creation of the database, its recalibration, alerts on modifications of the infrastructures and the constant precision in position estimation using an assailable measure of the RSSI, are always in the study [13,14,15].

In this study, we are extending our approach from static radio maps generation [12] to the dynamic and effortless technique using Wi-Fi-compatible IoT sensors. Our system automatically estimates the propagation loss parameters from the online feedback of sensors deployed in the environment. Similarly, online radio maps are automatically calibrated at regular intervals using new updates. A complete system that can be implemented is presented, from the configuration to the positioning output. Our system works with simple raster maps of the environment containing only the information on the walls, instead of complex vector maps in which each length and angle must be calculated accurately. Results show that the proposed system achieves a comparable accuracy in position estimation using IoT sensors versus a Wi-Fi survey-based positioning technique. The proposed system is completely effortless and does not require the intensive task of the survey of the environment to estimate the position of a mobile node.

The rest of the document is organized as follows: Section 2 describes details of some existing systems that help Wi-Fi fingerprinting to minimize survey efforts, Section 3 presents an introduction of the proposed system, while Section 4 describes the configuration and calibration of the sensors. Section 5 gives details of the pre-processing phase and Section 6 explains how RSSI maps are generated using configuration information. Then, the experimental setup, the results and discussions, and as well as the conclusion, are provided in Section 7, Section 8 and Section 9, respectively.

## 2. Existing Systems

### 2.1. Survey-Based Technique

Survey-based Wi-Fi fingerprinting technique consists of two phases: the offline phase of the database training survey and the online phase of location estimation. The training phase requires a comprehensive analysis of the environment to build an RSSI database with a certain degree of spatial granularity. It takes a lot of time to record the RSSI level at each point of the environment grid and becomes even more challenging in the working environments. Recently, many research efforts have been made to reduce this fastidious and time-consuming training phase of fingerprinting and several solutions have been proposed [2].

### 2.2. Interpolation-Based Systems

Some solutions using interpolation techniques with a modified path loss model at sparse reference points to reduce survey effort to a certain extent are also proposed. In [11], the author proposed a hybrid model that combines the fingerprint prediction model with a calibration procedure and provides comparable location accuracy using a few RSSI samples compared to the traditional method of fingerprinting. Triangular Interpolation and eXtrapolation (TIX) [10] uses only online Wi-Fi RSS values measured at each AP to obtain a linear mapping between RSS decay and distance, which further helps in estimating user’s location using the TIX algorithm. Similarly, a Signal-Distance Map (SDM) [16] works on the same principle, whereas the mapping of distance between the AP and RSS measurements is obtained by singular value decomposition (SVD).

### 2.3. Crowd-Sourcing-Based Techniques

Crowd-sourcing systems use active user inputs to create an online radio map database instead of using the tedious task of creating a training database through a survey. The Organic Indoor Localization OIL [17] integrates active user input after the training phase to extend the coverage and accuracy of fingerprint location in particular areas called Voronoi regions. EZ (easy) [18] does not require any prior information about the map and the location of the transmitter and resolves the environment, peripherals’ information, and distance parameters by running a genetic algorithm on RSS information reported by a background service running on mobile users. Walkie-Markie [7] also relaxes the prerequisite information of indoor RF infrastructures and attempts to generate maps of the environment using crowd-sourcing. The estimate of the Wifi-Mark in the Walkie-Markie requires a sufficient number of users who report the trajectory information of at least two sides of an AP. This is almost impossible from the point of view of the user and the architecture of the building, with corners and long continuous corridors in which it is generally not possible to move to the other side.

Recent research developments in ILBS technology use data from a wide range of sensors (inertial sensors, accelerometers, gyroscopes, magnetic sensors, etc.) to improve indoor positioning and mapping [19] using data fusion techniques [20,21,22,23]. WILL (Wireless Indoor Localization) and unLoc trace users’ movements using an inertial sensor to generate a map of the environment [6,9].

### 2.4. Probabilistic-Packet Transmission Technique

A probabilistic approach to location estimation using the fusion of multiple radio maps is described in the Probability Map Technique (PMT) [24]. The probability map technique uses RF packet transmission to estimate the RSSI value of a transmitting mobile node on multiple receivers. This technique is closely related to our static solution for radio map generation whereas it differs from our technique in the sense, that it uses probability distribution for distance calculation and uses a triangular density function in combination with a technique of geometric correction in the localization procedure.

### 2.5. Model-Based Systems

A model-based indoor positioning algorithm is presented in [25] to address the absorption effects of crowded scenarios on Wi-Fi signals through an indoor canyon environment and crowed sensing is achieved by using a mobile camera with the assistance of deep Convolutional Neural Network (CNN).

### 2.6. Vector-Map-Based Techniques

Indoor propagation prediction methods are generally divided into two categories. In the first approach, the free space propagation model is generated by fitting an empirical or statistical propagation to the measurement data. While the second approach is based on ray tracing in which the theory of electromagnetic propagation is applied. Here are some recent techniques that use vector maps of the environment to estimate indoor propagation.

#### 2.6.1. Empirical-Path Loss Technique

Empirical model based propagation predictions are simple in computation and are scalable. Here we discuss an enhanced empirical propagation model, which incorporates much of the propagation phenomena that are suggested by electromagnetic theory such as the uniform theory of diffraction (UTD). In this approach, propagation loss function has two distinct regions, the first region which is similar to free space and the second region where the effects of walls and ceiling become visible on propagation [26]. Each region has its own exponent of path loss. In addition, the dependence of the attenuation with an angle is introduced with the wall attenuation factor and the floor attenuation factor to adjust the loss of attenuation with respect to the incident angle.

#### 2.6.2. Deterministic-Ray Tracing and Radiosity Technique

In deterministic models, ray tracing and radiosity are very attractive due to their accuracy. However, due to the requirement of high computational and memory resources for 3D representation, these approaches are limited to 2D representation only. In [27], the author proposed a 2D approach to optimize the deterministic model of ray tracing by limiting the number of initial rays from the source by modeling the emitter as a circle and dividing it into horizontal elementary segments of the same length. In the case of radiosity [28], for each point of a scene, radiosity is defined as the sum of the radiation emitted plus the radiation reflected and transmitted at such a point from any other point of the scene.

There are also several other techniques proposed for automating propagation prediction to solve the tedious job of the manual survey of the environment. A closely related work is an online path-loss parameter estimation approach which is discussed in [29] where a variant of the particle filter (RBPF-AGB) is used to estimate the position of the target node. In [30], the author has proposed handling LOS and NLOS cases separately using the corresponding path loss model to enhance the positioning accuracy of indoor Wi-Fi environment. In [31], the author has proposed an approach to obtain the optimal deployment pattern of APs in the indoor environment to enhance the accuracy and reduce the calibration time required to survey the environment. The number of APs and their deployment locations are estimated using a technique based on a genetic algorithm that ensures that the quality of the radio map (RM) is not degraded during the generation process of RM. In addition, the radio maps are estimated by the Multi-Wall Extended (MWE) method using an empirically obtained WAF (Wall Attenuation Factor) value to minimize the effort of the survey required for fingerprint DB calibration.

Ray tracing seems to be an attractive solution, whereas, to ensure the rays incident at a certain point we need to increase the number of rays from emitting source which brings scalability issues due to high computational cost. Similarly, radiosity, with its high computational resources, limits the type of objects in the environment and their absorption and refraction properties to control the complexity of the maps in the calculation process. We have selected empirical method as a base model in our proposed solution due to its inherent characteristics of scalability, low computational cost and acceptance of an object of various types. And most importantly, the method based on ray tracing and radiosity requires vector maps while our system works with simple raster maps and requires no information on the angle and length of objects in the environment.

## 3. Proposed Approach

**Figure 1:**
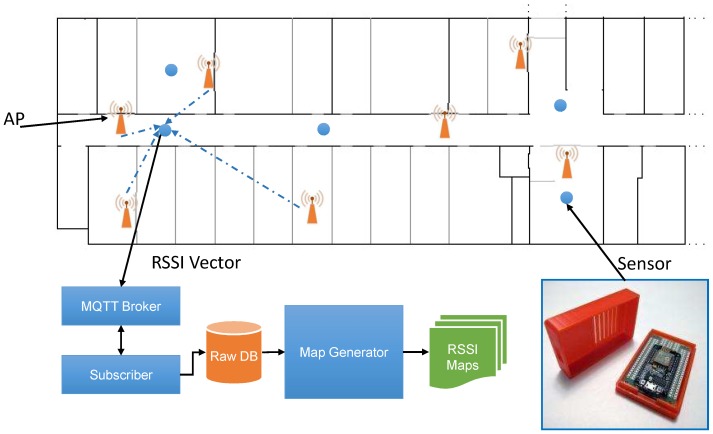
The system configuration diagram includes the sensors, APs; access points, and information flow from a sensor to the radio map generation engine.

In this study, we extend our approach of static path loss map generation using the raster map to more realistic dynamic online radio maps by adding IoT sensors to the environment, such as shown in Figure 1. The approaches discussed in the previous section are static in nature and generate maps only once using the permanent architecture of the environment (i.e., walls only). Considering that temporal changes and other short-term environmental changes are not addressed. To cope with such short-term changes in the Wi-Fi environment and provide a more realistic assessment of Wi-Fi signal propagation, we need active updates at particular locations in the environment. For such active updates, the proposed system uses IoT sensors equipped with Wi-Fi module as a scanning node uniformly distributed in the environment, as shown in Figure 1. Specifically, a sensor must be placed within 20 m of each AP to avoid parameter estimation based on the region [26]. We placed a sensor node at the center of two APs in a corridor and in selected rooms within the required range to develop proof of concept. These sensors analyze the environment after a defined period of time and send the RSSI scan information vector to an MQTT (Message Queuing Telemetry Transport) broker that publishes these updates to a database update subscriber. When the database subscriber receives updates from the MQTT broker, its job is to parse, format, and insert MQTT messages into the database. Next, the map generation engine collects the database information and using a pre-processed path loss template (metadata), generates a new radio map for each AP registered in the system. The radio maps are generated by estimating the RSSI at each cell of the 1m x 1m square grid on the physical surface.

Creating a raster map of the environment is a simple and important step in the proposed system. Pixel maps are created from the floor plans of the real-time test bench sites used in our experiments. First, the floor plan image is resized to a known resolution, e.g., 10 pixels/m. Then, each concrete wall with white lines (RGB 255, 255, 255) and wooden partitions with gray lines (RGB 128, 128, 128) is traced on a black background (RGB 0, 0, 0). For simplicity, only two types of walls are considered. The color value of the wall is an adjustment factor for the corresponding value of WAF induced. The adjustment factor must be carefully selected according to the thickness and material of the walls or obstacles. Finally, a grayscale image map with information on the size, type, orientation, and location of each wall in pixel format is obtained. Figure 1 shows an inverse view of the resulting map for a better view. In addition, the creation of maps and the relevant details have already been explained in our static approach to generating radio maps, so we ignore the further details and refer the reader to [12]. To set up the proposed system, after configuring the IoT sensors, two phases must be performed. Phase I, metadata generation of the path loss model that assists in the efficient generation of maps. Phase II, the online RSSI map generation phase uses the metadata produced during the pre-processing phase and uses new updates from sensors to generate radio maps of the environment for each selected AP. The detailed discussion of each phase is as follows.

## 4. Sensor Setup and Calibration

**Figure 2:**
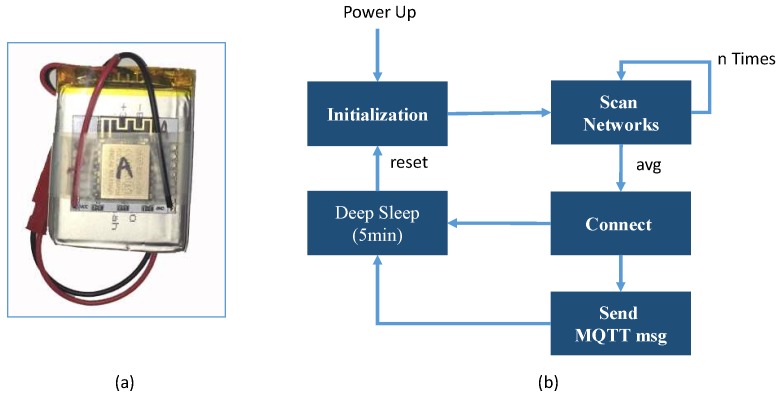
ESP8266 module as a Wi-Fi sensor node. (**a**) ESP8266 custom with battery, (**b**) Sensor operating cycle.

**Figure 3:**
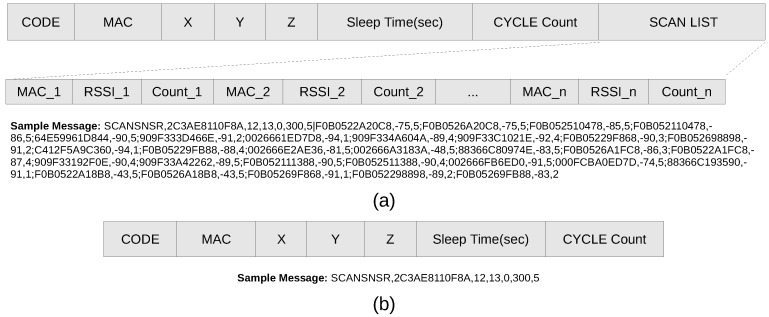
ESP8266 module configuration. (**a**) Received Signal Strength Indication (RSSI) scan message format. (**b**) Configuration update message format.

For active updates of Wi-Fi signal strength from certain points in the environment, an economical ESP8266 module that offers Wi-Fi capabilities as an RSSI scan sensor node is selected. Figure 2a shows the ESP8266 module used in our system and part (b) shows the operating cycle of the sensor as a scanning node. Depending on the work cycle, when the sensor is turned on, it scans the environment a specified number of times and averages the RSSI level of each AP in range. After scanning, the sensor format the information in the scan-message shown in Figure 3a. and sends it to the MQTT server before going to sleep for a specified time interval. The configuration of a node can be updated by sending a configuration message, as shown in Figure 3b. The configuration message of the sensor node includes the number of scans (sCount), the standby time in seconds (delay) and the position information (x, y, z).

**Figure 4:**
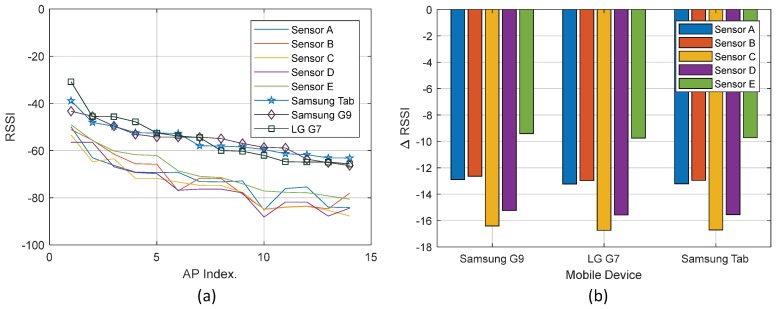
Calibration of Wi-Fi sensors. (**a**) Signal strength received from devices for the number of access points. (**b**) Signal difference in sensor power reception compared to mobile devices.

When the sensor starts sending updates to the system, this information can be used to calibrate the sensors. For sensor calibration, we simply calculate the RSSI reception difference of all sensors and the target mobile device. To this end, all the sensors and the target device are placed side by side, and the updates are saved. During this process, multiple scans of each sensor and mobile device are recorded to find the difference factor Δp. The difference factor for a particular device is used to correct the measured RSSI value in a scan message before it is stored in the database. A graphical representation of this calibration outcome is presented in Figure 4.

## 5. Offline Radio Map Metadata Generation (Phase I)

### 5.1. Access Points and Sensor Registration

**Figure 5:**
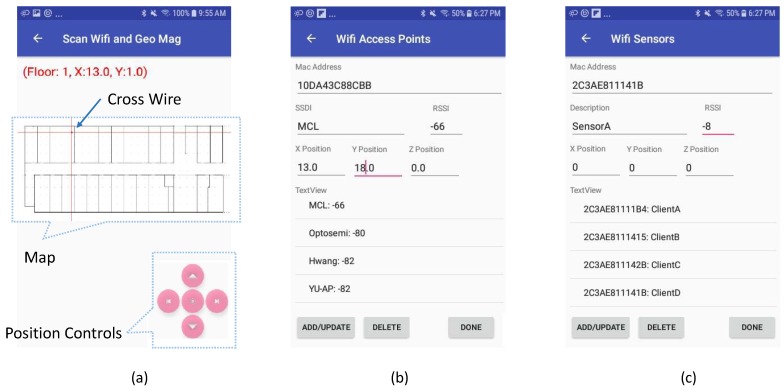
Smart-phone utility interfaces. (**a**) Positioning on Map, (**b**) Access point registration, and (**c**) Wi-Fi sensor registration.

The preprocessing phase begins with the registration of APs and sensors. In this process, the proposed system needs to collect the location and RSSI information of each AP, including $\left\{A,\;X,\;Y,\;P_{0}\right\}$, where *A* is the MAC address, XandY are the location parameters, and $P_{0}$ is the RSSI of AP at the distance of 1 m. Similarly, for each sensor, the proposed system requires {A,X,Y,Δp}, where *A* is the MAC address, XandY are the location parameters and Δp is the RSSI calibration parameter of Wi-Fi Module in the sensor. To configure sensors in the environment and register APs in the system, we have developed a mobile application that facilitates this process for a user to add the required information to the database. Figure 5 shows screenshots of three user interface forms used in the process. Figure 5a shows the image of the map. The map was created from the targeted site plan where indoor positioning is required. First, to register an AP in the system by using the map form, the user moves the cross-wires with the movement command buttons to the corresponding location of the AP and while remaining at a location where AP is deployed, press the middle button (Save Access Point button). When the user presses the middle button, the mobile device scans the environment five times and displays the average results on the AP interface shown in Figure 5b. In the list, the APs are ranked in order of highest to lowest RSSI. The user can select the corresponding AP from the list and save the information using the Add/Update button on the interface. In the same way, the user records the sensor information using the sensor interface form, as shown in Figure 5c. The result of this registration process is shown in Figure 1 where APs are marked in orange color, while the sensors are marked with blue circles.

### 5.2. WAF and Path Loss Constant Estimation

**Figure 6:**
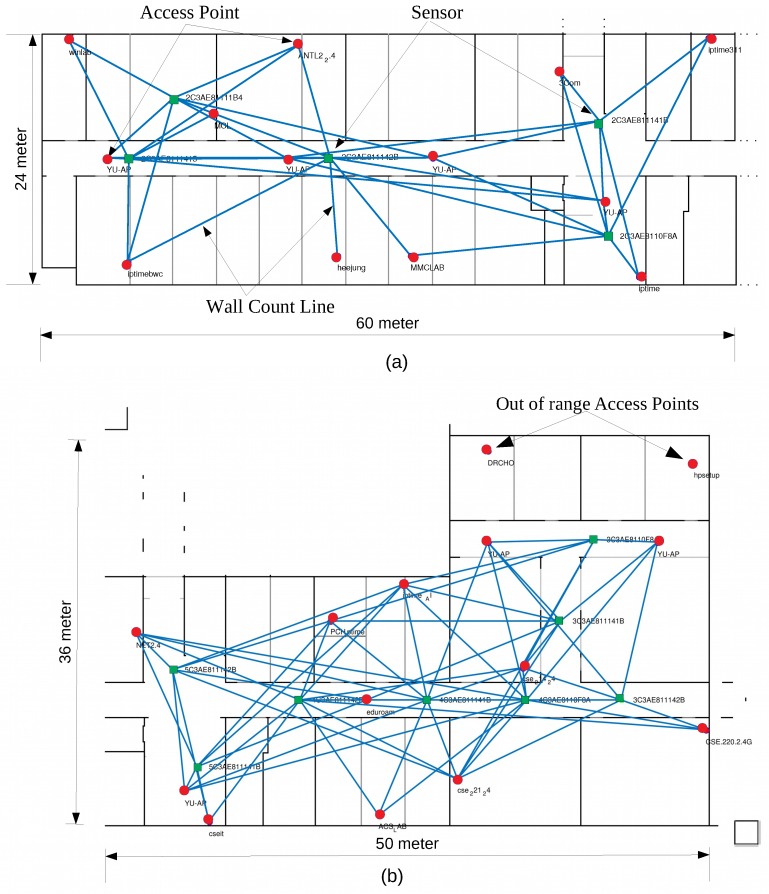
Experimental Setup. Sensors (green rectangles), access points (red circles), And the blue lines emerging from the sensors represent the distance of the access points present in the range of the corresponding sensor. (**a**) ICE Department and (**b**) CS Department.

The next step is to estimate the path loss constant of the environment and the attenuation factor of the walls. To this end, the proposed system uses the Particle Swarm Optimization (PSO) algorithm to estimate the two parameters [32]. Before executing the algorithm, we make sure to have a pixel map with a known resolution (i.e., pixels per meter) that we have created from floor plans of the building under experiment. On the maps, each concrete wall is represented by a white line (RGB 255, 255, 255) and a wooden partition by a gray line (RGB 128, 128, 128) on a black background (RGB 0, 0, 0). Doors and windows are also added with a gray color to display open and closed states to equalize the unpredictability error. The different pixel colors, ranging from 0 to 255 represents the range of materials in the environment. Here, two materials are presumed for the sake of simplicity. Figure 6 shows the lines emerging from each sensor to the corresponding visible APs at both sites. The pixel values beneath these lines help to count the number of walls as an abstraction between the sensor location and the AP location. Figure 6b presents a special case in which two APs are out of the reach of any sensor due to the low reception power of the sensor and the low transmission power of the access points (AP (DRCHO): −38 and AP (hpsetup): −42). Now, to calculate the WAF and the path loss constant (PLC), the following information is used:

(1) $$CostFunction_{Input}=MAP,RSSI,X_{AP_{i}},Y_{AP_{i}},P_{0},X_{S_{j}},Y_{S_{j}},X1,X2$$

where,

MAP is pixel map,

RSSI is the signal strength measure at sensor location,

$X_{{AP}_{i}},Y_{{AP}_{i}}$ are locations of AP,

$P_{0}$ is RSSI of AP at 1 m distance,

$X_{S_{j}},Y_{S_{j}}$ are locations of sensor,

X1 is value parameter for path loss constant,

X2 is value parameter for wall attenuation factor.

The information given in Equation (1) from each update is passed to the cost function of the PSO algorithm. The cost function is composed of Equation (1) to Equation (6).

(2) $$WallCount\left(w\right)=\frac{\sum maxima(GetPixelValueVector\left({\left(X,Y\right)}_{AP_{i}},{\left(X,Y\right)}_{S_{j}}\right))}{255}$$

(3) $$d_{S_{i}}=\frac{\sqrt{{\left(X_{{AP}_{i}}-X_{S_{j}}\right)}^{2}+{\left(Y_{{AP}_{i}}-Y_{S_{j}}\right)}^{2}}}{\Delta d}$$

(4) $$Estimated\;RSSI\left(LOS\right)=g\left(i,j\right)=P_{0_{i}}-10\times X1\times log\left(d_{S_{i}}\right)$$

(5) $$Estimated\;RSSI\left(NLOS\right)=h\left(i,j\right)=P_{0_{i}}-10\times X1\times log\left(d_{S_{i}}\right)-X2*w$$

(6) $$Cost=\left\{\begin{array}{cc} \sum RSSI(i,j)-g(i,j) & if\;w=0\;\;i.e.\;LOS\;Case\\ \sum RSSI(i,j)-h(i,j) & if\;w>0\;\;i.e.\;NLOS\;Case \end{array}\right.$$

**Figure 7:**
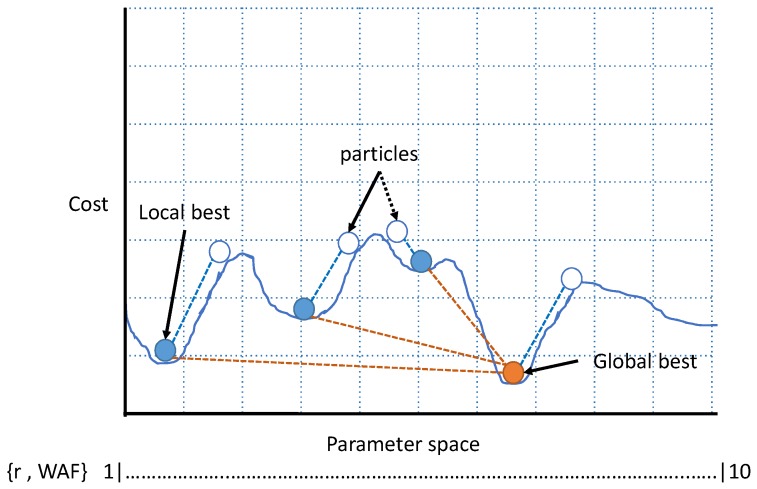
The estimation of parameter by Particle Swarm Optimization algorithm using value space vs. cost function output to find the global best solution.

PSO algorithm iterates values from 1 to 10 for the unknowns X1 and X2 as path loss constant *r* and wall attenuation factor WAF and generates the output as the best solution with the lowest cost, as shown in Figure 7. Instead of calculating the single-path loss constant for the whole site, the proposed system calculates separately for each case (LOS and NLOS) to a better control of the estimate of the propagation change in the environment.

### 5.3. Radio Map Metadata Generation

The last step of the first phase is the generation of metadata for the radio map of each AP. In this step, the proposed system defines the coverage area of each sensor and the corresponding AP on the site-map for generating radio maps. And most importantly, the preparation of metadata is done to reduce the computing resources required for Phase II, i.e., the generation of online RSSI maps. To generate a radio map of an AP at a resolution of 1 square meter, the proposed system needs the following information at each cell of the grid (x, y).

(7) $$\left\{MAC_{AP},P_{0},X,Y,d_{\left(i,j\right)},EPL_{AP},MAC_{S},d_{S_{i}},EPL_{S_{i}},RSSI_{S}\right\}$$

where $MAC_{AP}$ is MAC address of AP, $P_{0}$ is RSSI at 1 m distance from AP, (X,Y) are position coordinates of center of a grid cell,

(8) $$d_{\left(i,j\right)}=\frac{\sqrt{{\left(X_{{AP}_{i}}-X_{j}\right)}^{2}+{\left(Y_{{AP}_{i}}-Y_{j}\right)}^{2}}}{\Delta d}$$

(9) $$EPL_{AP}=\frac{\sum maxima(GetPixelValueVector\left({\left(X,Y\right)}_{AP_{i}},\left(X_{i},Y_{j}\right)\right))}{255}\times WAF$$

**Figure 8:**
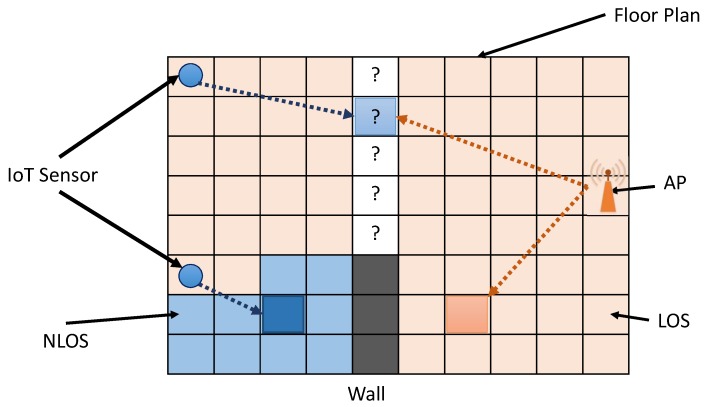
Selection of the contribution device for the estimation of RSSI. AP; Access point with the corresponding LOS; Line-of-sight region (light pink color) and the NLOS; NON-line-of-sight region (light blue color) which is the LOS region for the sensor. The white color cells with ‘?’ represent the regions that are in the LOS case for both kind of devices i.e., the sensor and the access point, and require the designation of a contributing device with respect to the minimal path loss.

**Figure 9:**
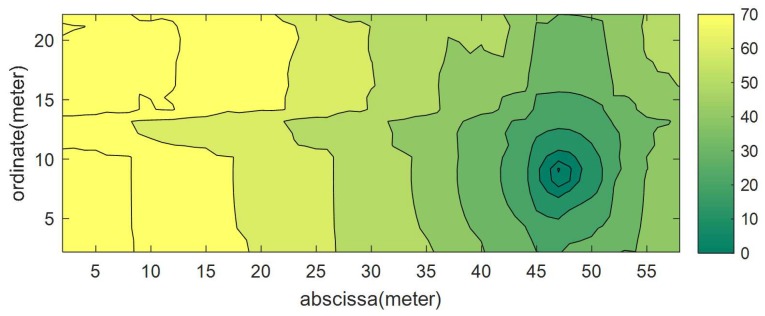
An estimated path loss map of a sensor.

**Figure 10:**
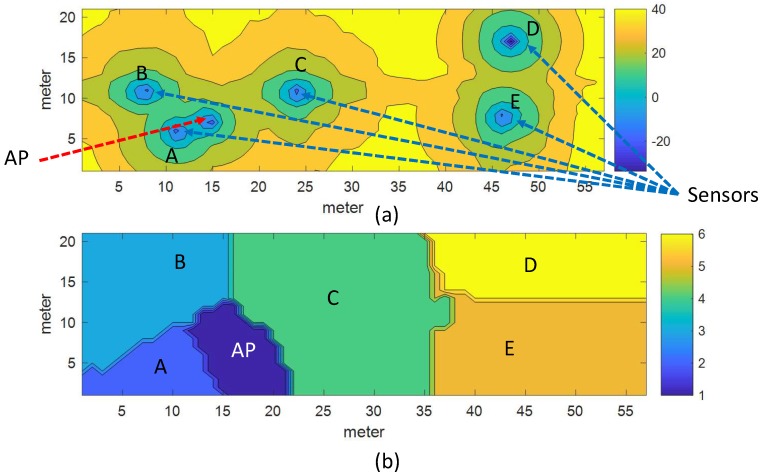
Zone allocation for Received Signal Strength Indication estimation on the basis of minimum path-loss (**a**) Combined path loss map of sensors and corresponding access point. (**b**) Calculated mask representing the device designation of a given region for the estimation of the radio map.

$MAC_{S},d_{S_{i}},EPL_{S_{i}},RSSI_{S}$ are parameters of a sensor i.e., MAC address, distance from AP, Effective Path Loss, and RSSI recorded by the sensor, respectively. These settings help the RSSI calculation at a particular grid cell. Figure 8 shows the problem of selection of the coverage area in the map estimation between AP and a sensor for points to the LOS criteria for both. There are many ways to choose the coverage area of each sensor in the radio map estimation of an AP, i.e., the shortest distance, the LOS and NLOS criteria, and minimal path loss. In this paper, the minimum path loss is considered. To select the contributing sensor in the RSSI estimate at a particular point, the proposed system calculates a path loss map for each sensor and the corresponding AP. Equations (8) and (9) help to calculate the path loss map, as shown in Figure 9. Similarly, the proposed system calculates path loss map $plMap_{AP}$ for each AP. Next Equation (10) shows the path loss map which is the combination of the minimum path loss at each cell of the grid, either by a sensor or by an AP. Equations (11)–(14) help find the $Mask_{S_{i}}$ that indicates the contribution area of each sensor radio map corresponding to the AP. Finally, the proposed system gets $Mask_{i}$ using Equation (15) that helps to identify the sensor or AP information to estimate the RSSI at a particular grid cell. It helps to optimize the IT resources required for the online phase of map generation. Figure 10a shows the combination of the minimum path loss and the part (b) represents the mask that defines the coverage area of the AP and sensors to participate in RSSI estimation. In the case of the AP, the RSSI estimation is made using the simple log to distance formula using its power at one meter, while in the case of the sensor, the RSSI estimation is made using the RSSI value present in the sensor’s feedback of a particular AP at the sensor location.

(10) $$plMap_{S_{i}}=\left[\begin{array}{cccc} pl(1,1) & pl(1,2) & \cdots & pl(1,n)\\ pl(2,1) & \ddots & \vdots \\ \vdots \\ pl(m,1) & \cdots & \cdots & pl(m,n) \end{array}\right]\;where\;\left(m,n\right)\;is\;size\;of\;grid$$

(11) $$pl\left(x,y\right)=10\times r\times log\left(d_{\left(x,y\right)}\right)+EPL_{\left(x,y\right)}$$

(12) $$plMap_{i}=argmin\left(plMap_{S_{1}},plMap_{S_{2}},\ldots ,plMap_{S_{n}},plMap_{AP_{k}}\right)$$

(13) $$Mask_{S_{i}}=ID_{S_{i}}\times U\left(plMap_{S_{i}},plMap_{i}\right)$$

(14) $$U\left(plMap_{S_{i}},plMap_{AP_{i}}\right)=\left\{\begin{array}{cc} 1 & plMap_{S_{i}}\left(x,y\right)=plMap_{AP_{i}}\left(x,y\right)\\ 0 & otherwise \end{array}\right.$$

(15) $$Mask_{i}=Mask_{S_{1}}+Mask_{S_{2}}+Mask_{S_{i}}+...+Mask_{S_{i}}+Mask_{AP_{i}}$$

## 6. Online RSSI Map Generation (Phase II)

The second phase begins when the proposed system has collected the information given in the Equation (7) for each 1-m square RSSI block (grid cell) on the map, except the last $RSSI_{S}$ value that is reported by a sensor at a particular time interval. This live RSSI feedback from each sensor on the APs present in the environment brings the online feature of radio maps generation that avoids the need to calibrate and recalibrate the RSSI database required for the fingerprinting technique. Equation (16) represents the radio map of an AP as a matrix of the function f(x,y) to m×n cells of the grid. The online RSSI estimation function is determined using the Equation (17).

(16) $$M\left(AP_{k}\right)=\left[\begin{array}{cccc} f(1,1) & f(1,2) & \cdots & f(1,n)\\ f(2,1) & \ddots & \vdots \\ \vdots \\ f(m,1) & \cdots & \cdots & f(m,n) \end{array}\right]\;where\;m=M/\delta ,\;n=N/\delta$$

(17) $$f\left(x,y\right)=\left\{\begin{array}{cc} P_{0}-10\times r\times log\left(d_{i}\right)-EPL_{AP} & L_{AP}<L_{S_{i}}\\ RSSI_{S_{i}}+L_{S_{i}}\left(x,y\right) & otherwise \end{array}\right.$$

(18) $$L_{S_{i}}\left(x,y\right)=10\times r\times log\left(d_{i}-d_{S_{i}}\right)+\left(EPL_{AP}-EPL_{S_{i}}\right)$$

Moreover, to further optimize the online computations required by f(x,y), the $MASK_{i}$ from Equation (16) helps in reducing the decision process specified in Equation (17) to select the sensor parameters for “otherwise” case. Finally using Equation (18), the fresh radio maps of selected APs are obtained to generate the fingerprinting database of a Wi-Fi environment for positioning purpose.

## 7. Experimental Setup

Two different office sites including Information and Communication Engineering (ICE) Department and Computer Science (CS) Department are selected as real-time test-bed for our experimentation with an area of $60\;\times \;24\;m^{2}$ and $50\;\times \;36\;m^{2}$, respectively, with ceiling height 3 m each. Each site area is divided at a resolution of 1 m × 1 m square per grid cell and 10 pixels/m on the pixel maps of both sites. Moreover, 13 and 14 APs permanently installed are selected in each environment and 5 and 8 sensors connected to the ceiling are deployed, respectively for the ICE and CS departments. Since the height of the ceiling at the two test sites is 3 m, the non-significant value of z-axis in our test environments, the z-axis is not considered in the calculation of the distance between the access point, the sensors and the reference points. However, for access points installed at high altitude, the z-axis must be taken into account for a correct calculation of the distance. Samsung Galaxy Tab2, Galaxy S9 + and LG G7 mobile devices are used for real-time data collection and position estimation. The Galaxy Tab2 device is used as the default target device. Fingerprinting Server is an Intel machine equipped with a Core i7 processor and 16 GB of RAM on Windows Server Edition with PostgreSQL database server.

## 8. Results and Discussion

### 8.1. Data Collection and Setup

**Figure 11:**
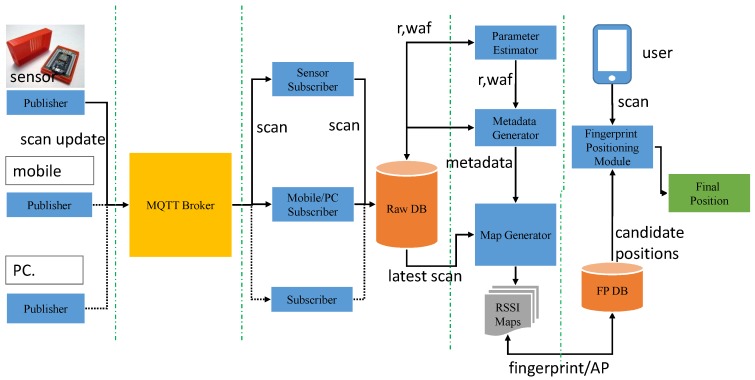
System flow diagram.

To begin the experiment, first, the sensors and target mobile devices are calibrated. All devices are placed side by side at the same location and 30 scan updates are collected, with each update averaging 5 scans. The RSSI level from 15 APs in the ascending order are compared, as previously reported in Figure 4. Table 1 shows the difference between sensors and mobile devices. Afterward, sensors are deployed into the environment and selected APs are registered in the system, as explained in Section 5.1. Each sensor sends information to the system every 5 min. The time-stamped feedback of the sensors is stored in the Raw DB using the MQTT broker, as shown in Figure 11.

Before the system begins to create the fingerprint database (radio maps), it estimates environmental pathway loss and the WAF as described in Section 5.2. For the estimation of these attenuation parameters, the feedbacks of the sensors during the hours when the displacements are less pedestrian are selected, for example, 12 a.m. to 1 a.m. This time selection is only a precaution to calculate the parameters in a static environment in the absence of any movement of people and logistics. When the parameter estimator completes its estimate, the metadata generator produces the metadata as explained in Section 5.3 using the estimated values of the propagation parameters. Finally, the map generator starts using the metadata generated for the day and the feedback of the sensors and generates a database of fingerprints for positioning purposes. In addition, the system continues to update the radio maps at the specified interval during working hours. Each update includes the parameters calculated for a particular day once in the first hour and new RSSI updates from deployed sensor locations. The minimum maps generation interval is directly proportional to the size of the environment, the number of APs, and the minimum time required for the map generation process. Table 2 shows statistics on map generation time for both sites using the experimental setup without any optimization of the programming. The statistics clearly show that the proposed technique requires relatively few computing and memory resources, as well as good scalability.

### 8.2. Path Loss and Attenuation Parameters Validation

**Figure 12:**
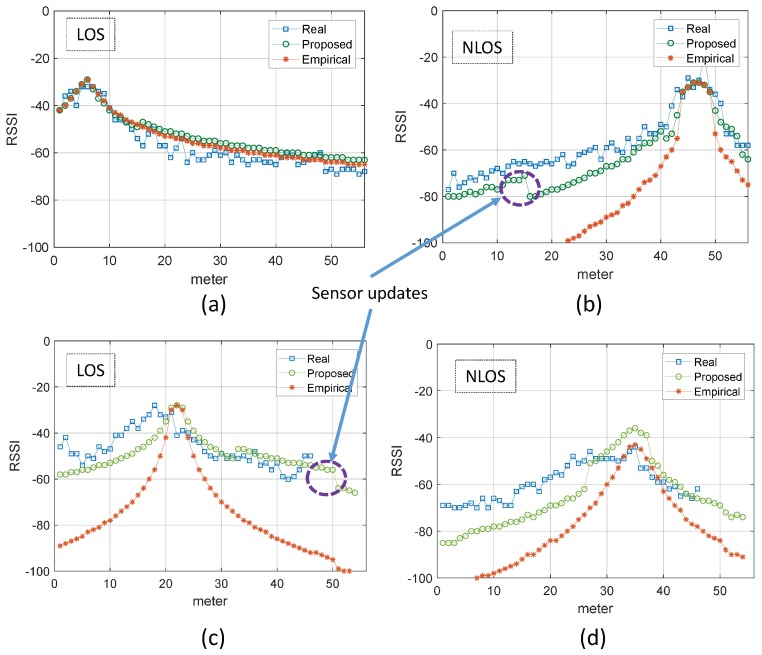
Comparison of Received Signal Strength Indication (RSSI) in real-time with the RSSI index estimated by the proposed system and the empirical technique of the two sites. (**a**–**d**) represent comparison for Access Points at LOS; Line-of-sight and NLOS; NON-line-of-sight regions for ICE and CS Departments, respectively. Sensor updates represents the correction of RSSI estimation from online feedback of corresponding sensor.

**Figure 13:**
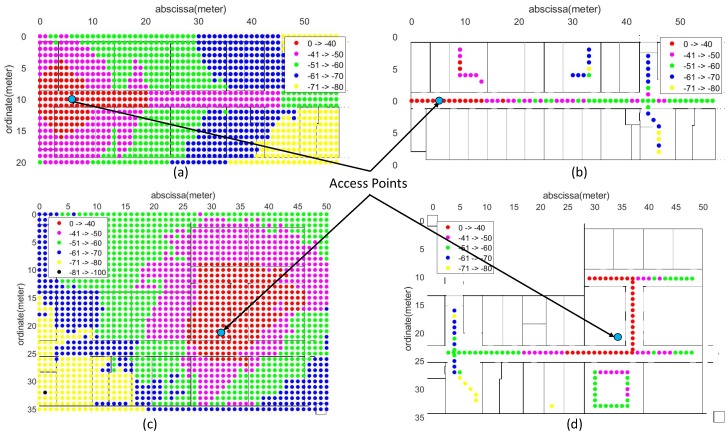
Comparison of 2-Dimensional radio maps of access points for both sites. (**a**) Estimated radio map and (**b**) Survey-based radio map for the ICE department. (**c**) Estimated radio map and (**d**) Survey-based radio map for CS departments.

To validate the evaluation of path loss and attenuation parameters, the RSSI values estimated from the proposed system and the empirical approach are compared with the actual RSSI values collected for both LOS and NLOS cases. The Figure 12 shows the comparison of some selected access points that remain in LOS or NLOS mode along a straight path in the corridor, part (a,c) and (b,d), respectively. For this validation and comparison purpose, the real-time data at 1 × 1 m^2^ grid points are collected on both sites. The graph clearly shows that the proposed system estimated RSSI values correlates better to the real time values in both cases as compare to estimation done using empirical values. The correction of the RSSI estimate using fresh sensor updates is highlighted using dark circles in Figure 12. Furthermore, to justify the accuracy of the radio maps generated using the automatically obtained environment parameters, compared to the radio maps generated using empirical parameters, the real-time RSSI values of each access point are compared to the corresponding estimated RSSI values of the empirical and proposed approach. The Table 3 summarizes the error statistics of the two techniques in the RSSI estimate against the real-time RSSI collected at particular grid points on both sites. Figure 13, shows the 2-Dimensional comparison of radio maps generated using the proposed approach and survey-based approach of selected APs for the Sites A and B, respectively. It is clear from the figure that the proposed approach works and allows the radio map to be estimated in places where access is restricted.

### 8.3. Map Validation

**Figure 14:**
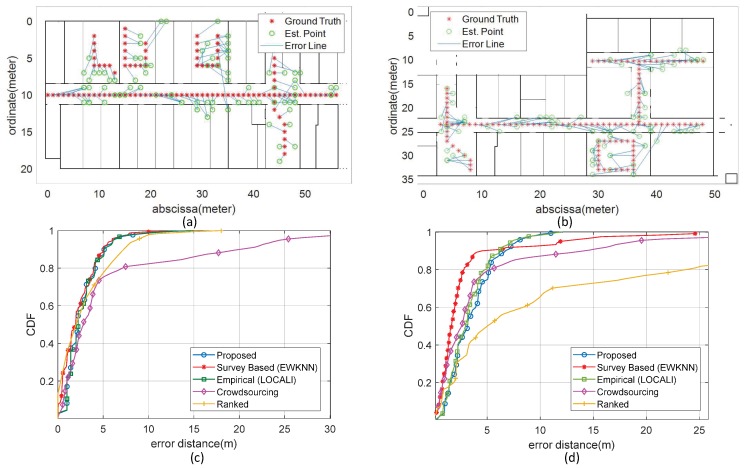
Comparison of ground truth, estimated position, and error distance for both sites. (**a**) The ICE department. (**b**) The CS department. And Cumulative distribution function of the positioning errors (CDF) for both sites. (**c**) The ICE department. (**d**) The CS department.

Finally, now to verify the accuracy of our radio maps, we generated the fingerprint database of both environments using the estimated RSSI values of the APs at each cell of 1 m × 1 m square grid. For the test, we selected a path that includes three lab rooms, a common room, a corridor and a stairway area for Site A, while for Site B, the selected path includes 3 corridors, a common room, and an area for stairs, as shown in Figure 14, Figure 14a,b as ground truth, respectively. Online scans are collected one meter apart along each path to verify the positioning accuracy of the system. The Table 4 shows the positioning accuracy statistics of the proposed system measured at 2nd quartile (50%), 3rd quartile (75%) and at a mean level compared to other systems. In addition, the standard deviation represents the uncertainty and the maximum error is also calculated for both sites. To the best of our knowledge the survey-based fingerprinting is the most accurate method of fingerprint database generation, therefore, Survey-based fingerprinting in combination with Enhanced Weighted K-Nearest Neighbour (EWKNN) algorithm [33] is selected to compare the position estimation accuracy of the proposed system. In addition, our static approach LOCALI [12] using empirically obtained values of r=2 and WAF=3.5 parameters, Crowdsourcing [6], and Ranked based [34] approaches are also included in comparison. Figure 14a,b show the ground truth, the estimated positions, and the corresponding error distance line, while parts (c) and (d) indicate the positioning error probability of the proposed system for sites A and B, respectively. The results of Table 4 and Figure 14 confirms the successful presentation of an effortless fingerprinting based positioning system, which has a precision comparable to that of a survey-based technique and surpasses performance by limiting deviation and maximum positioning error. Furthermore, the expected accuracy of our proposed system by exploiting the automatically obtained values of WAF and PLC parameters validates the correctness of the process of estimating parameter values.

### 8.4. Plug and Play Solution

**Figure 15:**
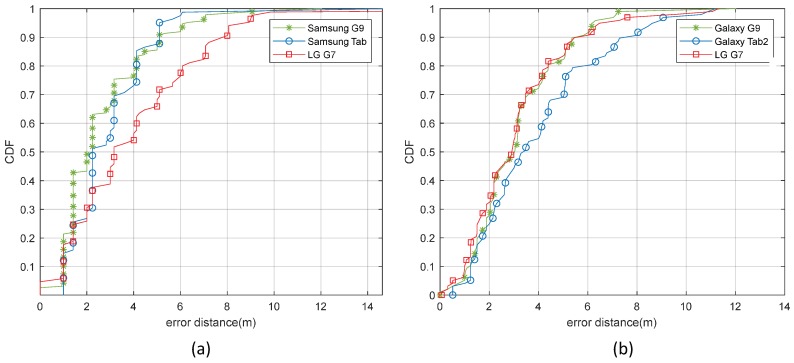
The probability of distance error in positioning for proposed system using different mobile devices at both sites. (**a**) The ICE department. (**b**) The CS department.

The strength of the proposed system lies in its “plug and play” features, works with simple raster maps and does not require detailed vector-based maps in which each length and angle must be calculated accurately. Secondly, the proposed system automatically estimates the propagation parameter of the environment and takes into account the temporal propagation loss through the online feedback of the sensors. Furthermore, changing Wi-Fi configurations, such as replacing, adding, or removing APs, requires a simple update of the registration list of APs instead of a recalibration survey of the complete environment. The correct configuration information of the location and power of the transmit/receive signal of the peripherals during the recording process may be considered a weakness of the proposed system. In Table 5, the proposed solution is compared with other automatic map generation solutions to highlight the advantages and disadvantages of the proposed approach. It is clear from the supported features that the proposed approach is more robust, practical in nature, and does not require significant computing resources or human effort to create radio maps. The proposed system works with simple raster maps and automatically estimates environment-specific attenuation parameters using IoT sensor online feedback. In addition, the positioning accuracy of the proposed system is also tested by using different mobile devices on the same paths at both sites and the data collection points that were followed previously while comparing the accuracy of the proposed system to other techniques. The results in Figure 15 show that the accuracy of the system in position estimation is consistent with different mobile devices.

## 9. Conclusions

In this paper, we investigated how the proposed system uses IoT sensors for automatic assessment of path loss and attenuation parameters in a Wi-Fi environment and for estimating signal propagation in a complex indoor environment by exploiting the architecture information provided in simple raster maps. In this way, the proposed system generates and updates the fingerprint database to enable indoor position estimation for fingerprint matching algorithms. In addition, a complete system that works from registration of devices and acquisition of online feedback from IoT sensors to the generation of the fingerprinting database is presented without any survey of the whole environment, in particular for an office like environments. Results show that the proposed technique is successful in automatic generation and calibration of the fingerprinting database and provides comparable accuracy in position estimation without doing any tedious job of the survey of the real environment. Possible directions for future work are, automated registration of AP with the location, radiant power of AP at 1 m, and directional path loss parameter assessment to improve the accuracy of fingerprinting. Incorporating deep learning based estimation of the propagation of the electromagnetic signal is another direction. 

## Figures and Tables

**Table 1:** Difference of received signal strength of mobile devices and that of sensors in dBm.

Device Name	Sensor A	Sensor B	Sensor C	Sensor D	Sensor E
Samsung Galaxy Tab2	−13.21	−12.95	−16.72	−15.55	−9.72
Samsung Galaxy S9 +	−12.90	−12.64	−16.41	−15.24	−9.40
LG G7	−13.23	−12.97	−16.74	−15.57	−9.74

**Table 2:** Statistics of area of sites, AP; number of access points, Sensor; number of sensors, $T_{PE}$; parameter estimation time, $T_{MG}$; metadata generation time, $T_{MU}$; map update time on sensor feedback and Map Size; map file size in MBs.

Building Type	Area (m${}^{2}$)	AP	Sensor	$T_{\mathrm{PE}}$ (s)	$T_{\mathrm{MG}}$ (s)	$T_{\mathrm{MU}}$ (s)	Maps Size
ICE Department	20 × 54	13	5	6	129	24	1.2 MB
CS Department	36 × 55	14	7	7	260	13	2.2 MB

**Table 3:** The difference error statistics in the RSSI values estimated using the proposed technique and the empirical technique against the real-time RSSI values collected at both sites. Site A (ICE Department Site), and Site B (CS Department Site).

	Site A		Site B	
	**Proposed**	**Empirical**	**Proposed**	**Emperical**
Mean error (dBm)	3.25	16.03	1.39	22.25
Min error (dBm)	−16	−9	−19	−13
Max error (dBm)	15	49	18	46
Standard deviation (dBm)	6.08	13.24	8.19	10.46
Correlation coefficient	0.95	0.84	0.82	0.77

**Table 4:** Positioning accuracy statistics, Std. Dev.; Standard Deviation and Max Error; maximum error for proposed system compared to other techniques at both sites.

Site	Technique	50%	75%	Average	Std. Dev.	Max Error
ICE Dept.	Proposed	2.23	3.59	2.06	2.71	15.00
	Survey-based (EWKNN)	1.95	3.35	2.38	2.18	15.0
	Empirical (LOCALI)	2.23	3.92	2.03	2.04	15.00
	Crowdsourcing	2.78	4.90	6.09	8.43	42.02
	Ranked	2.00	4.50	3.37	3.11	18.00
CS Dept.	Proposed	3.13	5.08	3.55	2.23	12.14
	Survey-based (EWKNN)	1.61	2.56	2.88	4.33	24.71
	Empirical (LOCALI)	2.96	4.27	3.31	2.13	12.03
	Crowdsourcing	2.58	4.00	4.86	7.49	45.00
	Ranked	5.00	18.00	12.05	14.22	49.06

**Table 5:** Qualitative comparison of the proposed system with other radio map generation techniques.

Features	Proposed	Empirical [26]	Ray Tracing [27]	Radiosity [28]	Low Efforts [31]
Support automated radio map generation	✓	✓	✓	✓	✓
Works with vector maps of environment			✓	✓	✓
Work with raster maps of environment	✓				
Support estimation of attenuation parameters	✓				
Support periodic updates	✓				
Works with existing AP deployment	✓	✓	✓	✓	
Requirement of high end computing resources			✓	✓	
Requirement of additional H/W (Sensors)	✓				

## References

[1] Liu H., Darabi H., Banerjee P., Liu J. (2007). Survey of wireless indoor positioning techniques and systems. IEEE Trans. Syst. Man Cybern. Part C (Appl. Rev.).

[2] Hossain A.M., Soh W.S. (2015). A survey of calibration-free indoor positioning systems. Comput. Commun..

[3] Chan Y.T., Ho K. (1994). A simple and efficient estimator for hyperbolic location. IEEE Trans. Signal Process..

[4] Bahl P., Padmanabhan V.N. RADAR: An in-building RF-based user location and tracking system. Proceedings of the INFOCOM 2000, Nineteenth Annual Joint Conference of the IEEE Computer and Communications Societies.

[5] Husen M.N., Lee S. (2016). Indoor Location Sensing with Invariant Wi-Fi Received Signal Strength Fingerprinting. Sensors.

[6] Yang S., Dessai P., Verma M., Gerla M. FreeLoc: Calibration-free crowdsourced indoor localization. Proceedings of the INFOCOM 2013.

[7] Shen G., Chen Z., Zhang P., Moscibroda T., Zhang Y. Walkie-Markie: Indoor pathway mapping made easy. Proceedings of the 10th USENIX Conference on Networked Systems Design and Implementation.

[8] Zhou B., Li Q., Mao Q., Tu W., Zhang X., Chen L. (2015). ALIMC: Activity landmark-based indoor mapping via crowdsourcing. IEEE Trans. Intell. Transp. Syst..

[9] Wu C., Yang Z., Liu Y., Xi W. (2013). WILL: Wireless indoor localization without site survey. IEEE Trans. Parallel Distrib. Syst..

[10] Gwon Y., Jain R. Error characteristics and calibration-free techniques for wireless LAN-based location estimation. Proceedings of the Second International Workshop on Mobility Management & Wireless Access Protocols.

[11] Narzullaev A., Park Y. (2013). Novel calibration algorithm for received signal strength based indoor real-time locating systems. AEU-Int. J. Electron. Commun..

[12] Ali M.U., Hur S., Park Y. (2017). Locali: Calibration-free systematic localization approach for indoor positioning. Sensors.

[13] Honkavirta V., Perala T., Ali-Loytty S., Piché R. A comparative survey of WLAN location fingerprinting methods. Proceedings of the WPNC 2009, 6th Workshop on Positioning, Navigation and Communication.

[14] Minaev G., Visa A., Piché R. Comprehensive survey of similarity measures for ranked based location fingerprinting algorithm. Proceedings of the 2017 International Conference on Indoor Positioning and Indoor Navigation (IPIN).

[15] Torres-Sospedra J., Richter P., Mendoza-Silva G., Lohan E.S., Huerta J. Characterising the Alteration in the AP Distribution with the RSS Distance and the Position Estimates. Proceedings of the 2018 International Conference on Indoor Positioning and Indoor Navigation (IPIN).

[16] Lim H., Kung L.C., Hou J.C., Luo H. (2010). Zero-configuration indoor localization over IEEE 802.11 wireless infrastructure. Wirel. Netw..

[17] Park J.G., Charrow B., Curtis D., Battat J., Minkov E., Hicks J., Teller S., Ledlie J. (2010). Growing an Organic Indoor Location System. Proceedings of the 8th International Conference on Mobile Systems, Applications, and Services.

[18] Chintalapudi K., Padmanabha Iyer A., Padmanabhan V.N. Indoor localization without the pain. Proceedings of the Sixteenth Annual International Conference On Mobile Computing and Networking.

[19] Khoshelham K., Zlatanova S. (2016). Sensors for indoor mapping and navigation. Sensors.

[20] Chen Z., Zou H., Jiang H., Zhu Q., Soh Y.C., Xie L. (2015). Fusion of WiFi, smartphone sensors and landmarks using the Kalman filter for indoor localization. Sensors.

[21] Hilsenbeck S., Bobkov D., Schroth G., Huitl R., Steinbach E. Graph-based data fusion of pedometer and WiFi measurements for mobile indoor positioning. Proceedings of the 2014 ACM International Joint Conference on Pervasive and Ubiquitous Computing.

[22] Li W.W., Iltis R.A., Win M.Z. A smartphone localization algorithm using RSSI and inertial sensor measurement fusion. Proceedings of the 2013 IEEE Global Communications Conference (GLOBECOM).

[23] Deng Z.A., Hu Y., Yu J., Na Z. (2015). Extended kalman filter for real time indoor localization by fusing WiFi and smartphone inertial sensors. Micromachines.

[24] Knauth S., Ortega A.A.B., Dastageeri H., Griese T., Tran Y. Towards Smart Watch Position Estimation Employing RSSI based Probability Maps. Proceedings of the First BW-CAR Baden-Württemberg CAR Symposium on Information and Communication Systems (SInCom 2014).

[25] Jiao J., Li F., Deng Z., Ma W. (2017). A smartphone camera-based indoor positioning algorithm of crowded scenarios with the assistance of deep CNN. Sensors.

[26] Cheung K.W., Sau J.M., Murch R.D. (1998). A new empirical model for indoor propagation prediction. IEEE Trans. Veh. Technol..

[27] Ayadi M., Torjemen N., Tabbane S. (2015). Two-Dimensional Deterministic Propagation Models Approach and Comparison With Calibrated Empirical Models. IEEE Trans. Wirel. Commun..

[28] Belmonte-Fernández Ó., Montoliu R., Torres-Sospedra J., Sansano-Sansano E., Chia-Aguilar D. (2018). A radiosity-based method to avoid calibration for indoor positioning systems. Expert Syst. Appl..

[29] Bruno L., Addesso P., Restaino R. (2014). Indoor positioning in wireless local area networks with online path-loss parameter estimation. Sci. World J..

[30] Bose A., Foh C.H. A practical path loss model for indoor WiFi positioning enhancement. Proceedings of the 2007 6th International Conference on Information, Communications & Signal Processing.

[31] Ficco M., Esposito C., Napolitano A. (2014). Calibrating Indoor Positioning Systems with Low Efforts. IEEE Trans. Mob. Comput..

[32] Clerc M., Kennedy J. (2002). The particle swarm-explosion, stability, and convergence in a multidimensional complex space. IEEE Trans. Evol. Comput..

[33] Shin B., Lee J.H., Lee T., Kim H.S. Enhanced weighted K-nearest neighbor algorithm for indoor Wi-Fi positioning systems. Proceedings of the 2012 8th International Conference on Computing Technology and Information Management (ICCM).

[34] Machaj J., Brida P., Piché R. Rank based fingerprinting algorithm for indoor positioning. Proceedings of the 2011 International Conference on Indoor Positioning and Indoor Navigation (IPIN).

